# Vocal cord dysfunction presented as difficult to control Asthma

**DOI:** 10.5339/qmj.2023.sqac.26

**Published:** 2023-11-26

**Authors:** Sara Mohamed, Hassan Mobayed

**Affiliations:** ^1^Allergy & Clinical Immunology division, Department of Medicine, Hamad Medical Corporation, P.O. Box 3050, Doha, Qatar Email: SMohamed54@hamad.qa

## Introduction

Vocal cord dysfunction (VCD) can result from several psychological conditions such as stress, anxiety, and posttraumatic stress disorder; however, chronic gastroesophageal reflux disease (GERD) can cause it. The Clinical picture can mimic severe asthma and limit the patients quality of life. Treatment of vocal cord dysfunction can lead to complete resolution of the symptoms.

## Case Presentation

A 42-year-old woman was diagnosed with asthma based on her symptoms. She was maintained on Fluticasone furoate/Vilanterol 200/25 mcg daily. Still, her symptoms of cough, difficulty breathing, and hoarseness of voice were not controlled, so she was referred to the severe asthma clinic. Her initial investigations showed normal spirometry with no reversibility after bronchodilator, low eosinophil count (200 /l), low total IgE (14 kunits/L), and low FENO (6.5 ppb). Symptoms were not typical for asthma, and the presence of hoarseness of voice, especially on exercise, was alarming, so the diagnosis of asthma was not solid. She was referred to ENT for assessment of VCD. Flexible laryngopharyngeal showed acid reflux. She received some instructions and medical treatment for GERD and was referred to speech therapy, where she was trained on vocal cord relaxation techniques. Within a few weeks, she showed dramatic improvement in her symptoms, and she could step down her asthma medications till discontinued. A Methacholine challenge test was done while she was off asthma medications for a few months and was negative, excluding asthma. C.T. scan of chest and neck was normal. The patient has been off asthma medications for over a year and is free of symptoms.

## Conclusion

VCD is the closure of the vocal cords during inspiration causing partial airway obstruction. It can affect about 25% of patients. Patients can present with stridor, wheezing, or dysphonia. Many factors, including GERD and postnasal drip, can trigger VCD. VCD can mimic severe asthma; therefore, many patients are treated with high-dose ICS; some patients even started on biologics. VCD due to GERD is among the differential diagnosis of severe asthma. The first-line therapy is breathing maneuvers and vocal cord relaxation techniques. Clinicians must confirm the diagnosis and exclude asthma mimics when treating severe asthma.

Conflict of Interest: we have no conflict of interest.
